# Consequences of negative energy balance on follicular development and oocyte quality in primiparous sows^†^

**DOI:** 10.1093/biolre/ioz175

**Published:** 2019-09-02

**Authors:** N G J Costermans, K J Teerds, A Middelkoop, B A J Roelen, E J Schoevers, H T A van Tol, B Laurenssen, R E Koopmanschap, Y Zhao, M Blokland, F van Tricht, L Zak, J Keijer, B Kemp, N M Soede

**Affiliations:** 1 Human and Animal Physiology, Wageningen University and Research, Wageningen, The Netherlands; 2 Adaptation Physiology, Wageningen University and Research, Wageningen, The Netherlands; 3 Faculty of Veterinary Medicine, Utrecht University, Utrecht, The Netherlands; 4 Wageningen Food Safety Research (WFSR), Wageningen University & Research, Akkermaalsbos 2, 6708WB Wageningen, The Netherlands; 5 TopigsNorsvin Research Center B. V., Beuningen, The Netherlands

**Keywords:** follicle, follicular development, in vitro maturation (IVM), in vitro fertilization (IVF), gonadal steroids, estradiol, insulin-like growth factor, lactation, metabolism, porcine, zygote

## Abstract

Metabolic demands of modern hybrid sows have increased over the years, which increases the chance that sows enter a substantial negative energy balance (NEB) during lactation. This NEB can influence the development of follicles and oocytes that will give rise to the next litter. To study effects of a lactational NEB on follicular development, we used 36 primiparous sows of which 18 were subjected to feed restriction (3.25 kg/day) and 18 were full-fed (6.5 kg/day) during the last 2 weeks of a 24.1 ± 0.3 day lactation. Feed restriction resulted in a 70% larger lactational body weight loss and 76% higher longissimus dorsi depth loss, but similar amounts of backfat loss compared to the full fed sows. These changes were accompanied by lower plasma insulin-like growth factor 1 (IGF1) and higher plasma creatinine levels in the restricted sows from the last week of lactation onward. Ovaries were collected 48 h after weaning. Restricted sows had a lower average size of the 15 largest follicles (−26%) and cumulus–oocyte complexes showed less expansion after 22 h in vitro maturation (−26%). Less zygotes of restricted sows reached the metaphase stage 24 h after in vitro fertilization and showed a higher incidence of polyspermy (+89%). This shows that feed restriction had severe consequences on oocyte developmental competence. Follicular fluid of restricted sows had lower IGF1 (−56%) and steroid levels (e.g., β-estradiol, progestins, and androgens), which indicated that follicles of restricted sows were less competent to produce steroids and growth factors needed for oocytes to obtain full developmental competence.

## Teaser

**Summary sentence**: Premating feed restriction results in lower oocyte developmental competence, which might be explained by reduced follicular steroid and growth factor production.

## Introduction

The metabolic state of a female can influence reproductive outcome, which is especially evident in highly productive animal species, such as pigs. Sows usually experience a negative energy balance (NEB) during lactation due to high metabolic demands for milk production, while feed intake is not sufficient to cover the demands for milk production and maintenance [1]. Over the last decades, metabolic demands of modern hybrid sows have increased as litter size and number of piglets weaned have risen. Consequently, sows have a higher chance to enter a substantial NEB during lactation, especially first parity sows that still have a low feed intake capacity [2]. This premating NEB can negatively affect reproductive outcome when weight loss exceeds 10%–12% of initial body weight [3] and is especially evident in primiparous sows leading to reduced second parity reproductive performance [4,5]. High lactational weight loss can lead to a reduction in farrowing rate and litter size [3,6]. In addition, negative influences on embryonic development and litter uniformity [7,8] and subsequent variation in piglet birth weights [9] have been reported. The exact origin of reduced reproductive outcome after NEB remains for a part unknown.

The negative effects of the lactational NEB on reproductive outcome can be partly explained by the influence of the premating metabolic state, during and after lactation, on the development of follicles from which oocytes will give rise to the next litter as reviewed by Prunier and Quesnel, 2000 [10]. For instance, feed restriction during lactation resulted in a smaller follicle size at weaning and 48 h postweaning [11] and decreased oocyte maturation rates when oocytes were isolated 38 h before the anticipated onset of oestrus [12]. Nevertheless, research investigating relations between the premating metabolic state and follicular and oocyte competence in modern hybrid sows, which experience higher metabolic demands during lactation, is lacking.

The aim of this study is to determine effects of lactational feed intake level and metabolic state on postweaning follicular and oocyte developmental competence in modern hybrid sows. This was done by subjecting sows to feed restriction during the last 2 weeks of their first lactation. The lactational metabolic state was assessed as well as follicular and oocyte developmental competence 48 h after weaning.

## Materials and methods

### Ethics statement

The experiment was approved by the Animal Care and Use Committee of Wageningen University (DEC2017048) and performed in accordance with national, EU and SSR's specific guidelines and standards at research facility CARUS (Wageningen University & Research, the Netherlands).

### Animals

A total of 36 TN70 (Large White × Norwegian Landrace, TopigsNorsvin, Vught, The Netherlands) gilts originated from a single gilt breeding farm in three consecutive batches. The gilts arrived 2 weeks before farrowing at an average age of 363 ± 21 days and were divided over two farrowing rooms. During the final 2 weeks of gestation, sows were fed twice a day (07.00 and 16.00 h) and received 2.9 kg/day of a standard gestation diet (standard sow feed, AgruniekRijnvallei, Wageningen, The Netherlands). Within 72 h after parturition, piglets were cross-fostered to obtain similar litter sizes. During lactation, sows were fed an allocated amount of standard lactation diet (ca. 9.3 MJ NE/kg, 156 g/kg CP, 8.9 g/kg lysine, 7.69 SID lysine; Maxima lacto, AgruniekRijnvallei) divided into three meals a day (07.00, 13.00, and 19.00 h). All sows received the same amount of the lactation diet until 2 weeks before weaning, starting at 2 kg/day at parturition and increasing with around 0.5 kg/day to reach 6 kg/day 2 weeks before weaning, according to the TN70 feeding manual for primiparous sows (TopigsNorsvin). During the last 2 weeks of lactation, sows were either full-fed (FF; 6.5 kg/day; *N* = 18) or restricted-fed (RES; 3.25 kg/day; *N* = 18). Their piglets were creep-fed. Sows were allocated to treatments based on body weight, backfat thickness, and muscle depth at parturition, and treatments were equally distributed over both farrowing rooms. Sows had an average lactation length of 24.1 ± 0.3 days for both FF and RES, and weaned 12.4 ± 0.6 vs. 12.3 ± 0.6 piglets for FF and RES, respectively. From the day of weaning, all sows received a similar amount of the lactation diet (3 kg/day) until 48 h after weaning, after which the sows were slaughtered by stunning and exsanguination.

### Blood sampling

Blood samples of overnight fasted sows were taken at the first day of lactation (D1), 2 weeks after the start of the feed restriction period (D17), at weaning (D24) and at slaughter (D26), between 06.00 and 07.00 h. The sows were restrained with a nose-sling, and blood was collected from the jugular vein in 9 mL serum clot activator tubes and in 9 mL EDTA coated tubes (Greiner Bio-One, Monroe, NC) to obtain serum and plasma samples, respectively. The collection tubes were stored on ice. After blood sampling, the EDTA tubes were immediately centrifuged, while the serum tubes were first incubated for 1 h at 4 °C. Centrifugation took place at 3000×*g* for 10 min at 4 °C. Both plasma and serum samples were stored at −20 °C until further analysis.

### Body weight, backfat depth, muscle depth

All sows were weighed and P2 backfat depth and loin muscle depth were measured at arrival (D-16), D1 of lactation, at the start of the feed restriction period (D10), at D17, and at D24 (weaning). Body weight was additionally measured at D26 (slaughter). Backfat and loin muscle depth were measured in triplicate at the right and left P2 positions, which is located 6 cm from the midline, at the position of the last rib using B-mode ultrasonography with a 6.5 MHz linear transducer (Aquilla, Esaote, Genova, Italy), similar to Hoving *et al.* [13]. Piglets were weighed within 24 h after parturition and at weaning.

### Follicle size

Immediately after slaughter, the left and right ovary were placed against a ruler and photographed from three different sides, to facilitate measurement of the follicle size of all visible antral follicles. Follicle size was determined as the largest macroscopically visible diameter of the follicle and measured from the photographs using ImageJ (version 1.51f, National Institutes of Health, Bethesda, USA).

### Follicular fluid and COC collection

The right ovary was immediately frozen in liquid nitrogen and stored at −80 °C until further analysis. Left ovaries were placed in plastic bags in a water bath at 37 °C. The 15 largest follicles of the left ovary were aspirated within 5 h after collection, using a 21G × 5/8 needle and 1 mL syringe (VWR, Amsterdam, The Netherlands). These are assumed to represent approximately half of the ovulatory follicle pool, as ovulation rates in modern sows are around 25 [14]. After recovery of the cumulus–oocyte complexes (COCs), the follicular fluid content of the 15 largest follicles was pooled, collected in a tube and allowed to settle for 5 min. The supernatant was removed and centrifuged at 1900×*g* at 4 °C for 30 min to separate cells from the follicular fluid. The total volume of follicular fluid was assessed by reverse pipetting and was subsequently stored at −80 °C until further analysis.

The recovered COCs were used for in vitro maturation (IVM) and in vitro fertilization (IVF). The COC recovery rate was 64.7 ± 5.6 vs. 72.2 ± 3.9 for RES and FF, respectively.

### IVM and IVF

IVM and IVF took place at 38.5 °C in a humidified atmosphere of 5% CO_2_ in air. All compounds used to make the media were obtained from Sigma-Aldrich, unless otherwise stated. All media were preincubated at least 2 h prior to use. The pool of COCs of each sow was separately collected and transferred to a one-eyed dish in IVM-wash medium, which consisted of NCSU-23 [15] supplemented with 0.6 mM cysteine, 1 mM l-glutamine, 25 μM β-mercaptoethanol, 2.2 mg/mL NaHCO_3_, 25 mM unbuffered HEPES, and 10% v/v porcine follicular fluid. This pool of porcine follicular fluid was formed by pooling follicular fluid collected from sows using antral follicles between 3 and 8 mm. Recovered COCs from each sow were transferred to a four-well culture dish, washed once, and cultured in IVM-I medium (M199, Gibco BRL, Paisley, UK) supplemented with 2.2 mg/mL NaHCO_3_, 10% v/v porcine follicular fluid, 5.0 mM sodium pyruvate, 1 mM l-glutamine, 200 μM cysteamine, 0.05 IU/mL Follicle-stimulating hormone (FSH), and 1% pen/strep. Photographs were taken for assessment of COC morphology and COC size. After 22 h, COCs were washed once and cultured for another 22 h in IVM-II medium (IVM-I medium without FSH). In this system, cumulus expansion was not measurable after 44 h as the cumulus cells showed a very high degree of expansion. After 44 h of IVM, COCs were denuded by repeated pipetting, washed once, and incubated for 30 min in Modified Tris-buffered medium (13.1 mM NaCl, 3.0 mM KCl, 20.0 mM Tris, 11.0 mM d-glucose, 7.5 mM CaCl_2_·2H_2_O, and 5.0 mM Na-pyruvate) containing 1 mM caffeine, 0.1% BSA, and 1% pen/strep. One milliliter of fresh extended semen originating from five different Tempo boars (TopigsNorsvin) was added to 3 mL of IVF medium and centrifuged at 700*×g* for 5 min at 25 °C. Supernatant was removed, and sperm was washed twice by resuspending the pellet in 8 mL of IVF medium, and centrifuged as described above. Sperm cells were added to the denuded oocytes to obtain a final concentration of 1 × 10^4^ sperm cells/mL. The sperm and oocytes were coincubated for 24 h.

### Presumptive zygote staining

The developmental stage of the presumptive zygotes was assessed 24 h after IVF using an immunofluorescent staining for 5-methylcytosine and propidium iodide to stain total DNA. The 5-methylcytosine staining was used to better distinguish developmental stages of the zygotes, as methylation patterns change during zygote development [16]. Presumptive zygotes were fixed in 4% paraformaldehyde for 15 min and incubated overnight in 1% paraformaldehyde +0.2% Triton. Subsequently, the presumptive zygotes were incubated in 4 N HCl for 15 min and neutralized in 100 mM Tris–HCl (pH 8.0) for 10 min. Zygotes were washed 3 × 5 min in PBS pH 7.4 (+0.1% w/v Polyvinylpyrrolidon (PVP)), blocked in 5% (v/v) normal goat serum in PBS (+0.1% w/v PVP) for 60 min and incubated with anti-5-mC mouse antibody (39649, cloner: 33D3, Active Motif, Carlsbad, CA) diluted 1:500 (v/v) in PBS-BSAc (Aurion, Wageningen, The Netherlands) for 60 min. Zygotes were washed 3 × 5 min in PBS (+0.1% PVP) and incubated with secondary goat antimouse Alexa Fluor 488 (A-21131, ThermoFisher Scientific, Waltham, MA) diluted 1:200 (v/v) in PBS-BSAc for 60 min at room temperature. After 3 × 5 min washing, zygotes were counterstained with propidium iodide (Sigma-Aldrich) diluted in PBS (1:100) for 5 min and mounted on microscope slides (Menzel-Gläser, Braunschweig, Germany) to assess the developmental stage and methylation status of the presumptive zygotes. Zygotes were imaged at 40 times magnification using a fluorescence microscope (Leica DM6B), a digital camera (DFC365 FX), and imaging software (LasX; all Leica Microsystems, Amsterdam, The Netherlands).

### IVM and IVF outcome

Photographs taken at 0 and 22 h after start of IVM were used for COC morphology analysis and COC size measurements. COC morphology was assessed at 0 h to determine the number of normal (healthy) and atretic (unhealthy) COCs for each sow, according to Alvarez et al. [17]. Individual COC size was determined and averaged for each sow using ImageJ (National Institutes of Health).

The percentage COC expansion was defined as the average COC size at 22 h divided by the average COC size at 0 h. The developmental stage of the presumptive zygotes was studied 24 h after IVF, and individual zygotes were classified according to pronuclear stages as described in Wossidlo et al. [16]. Classifications used were, pronuclear stage 0 (PN0), pronuclear stage 1–5 (PN1–5), metaphase, and G2 (2-cell stage). The percentage fertilized oocytes was defined for each sow by dividing the number of zygotes from the pronucleus stage onward by the total number of COCs. Percentage polyspermy was determined by dividing the number of fertilized zygotes with three or more pronuclei by the total number of zygotes. IVM and IVF results of two RES sows were excluded from analysis since fewer than five COCs were successfully isolated.

### Follicular fluid steroid profiling

A representative subset of 12 FF and 12 RES sows were chosen based on average weight loss during lactation and average follicle size and used for follicular fluid endogenous steroid hormone profiling. The pooled follicular fluid of the 15 largest follicles of the left ovary was used. A slightly modified sample cleanup and mass spectrometric detection method as described in Blokland et al. [18] was used to detect endogenous aglycons in follicular fluid. The sample cleanup consist of mixing 900 μL water with 100 μL follicular fluid after which the diluted sample is passed through an Oasis HLB 96-well solid phase extraction plate (Waters, Milford, CT). To enhance the sensitivity of the mass spectrometric method derivatization of aglycons was performed with picolinic acid. The aglycons were separated by chromatography on a Waters BEH C_18_ column (Waters) followed by analysis on a Xevo TQ-S mass spectrometer (Waters) in positive ESI mode.

**Figure 1:**
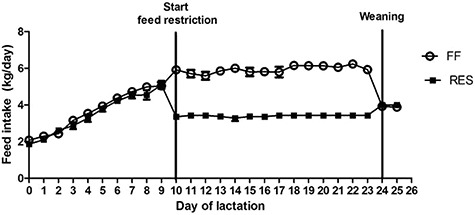
Average daily feed intake (kg/day) of full-fed (FF) and restricted-fed (RES) primiparous sows. All sows received a similar gradually increasing level of a standard lactation diet up to 6 kg/day at day 10 of lactation. From day 10 until weaning at day 24, sows either received 6.5 or 3.25 kg/day, respectively. After weaning, all sows received 4 kg/day of the lactation diet.

**Figure 2:**
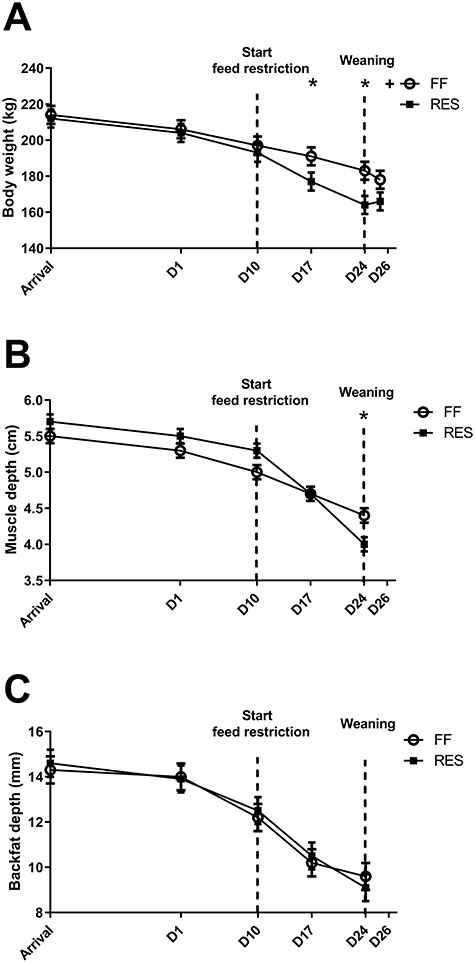
(A) Average body weight (kg), (B) longissimus dorsi muscle depth (mm), and (C) backfat depth (mm) for full-fed (FF) and restricted-fed (RES) primiparous sows, which received either 6.5 or 3.25 kg/day for the last 2 weeks of lactation. Body weight, loin muscle, and backfat depth were measured at arrival, the first day of lactation (D1), at the start of the feed restriction period (FR) (D10), 1 week after start of FR (D17) and at weaning (D24). Body weight was additionally measured at slaughter (D26). ^*^P-value <0.05, + *P*-value >0.05 and <0.10. Parameters were always significantly different between days within treatment (*P* < 0.05), except for muscle depth at arrival and D1 for the FF sows.

**Figure 3:**
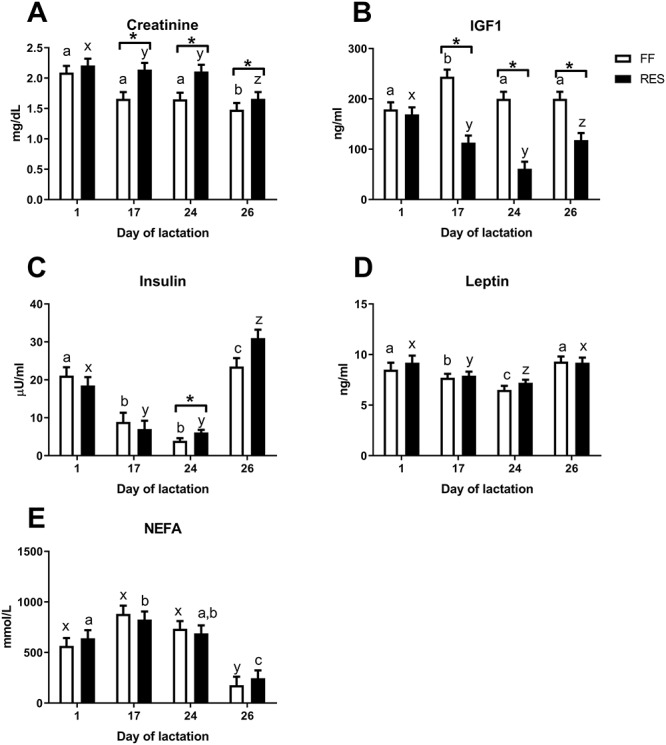
Fasted plasma (A) creatinine, (B) IGF1, (C) basal insulin, (D) leptin, and (E) serum NEFA levels of full-fed (FF), and restricted-fed (RES) primiparous sows, which received either 6.5 or 3.25 kg/day for the last 2 weeks of lactation. Measurements were done at the first day of lactation (D1), 1 week after start of the feed restriction period (D17), at weaning (D24) and at slaughter (D26). ^*^P-value < 0.05. The effect of measurement day for each parameter are depicted above the figures. Different letters indicate a significant difference between days within a treatment (abc for FF and xyz for RES).

### Blood serum and plasma measurements

All assay procedures were performed according to manufacturer’s instructions, unless stated otherwise. All analyses were performed in duplicate and only samples with an intra-assay CV ≤ 15% were included, which resulted in the exclusion of a few samples. Plasma insulin and leptin concentrations were measured using a radioimmunoassay kit (Porcine Insulin PI-12 K and Multi-Species Leptin XL-85 K, respectively, EMD Millipore Corporation, Billerica, MA). Plasma insulin-like growth factor 1 (IGF1) was measured with an immunoradiometric assay (A15729, Beckman Coulter, Woerden, The Netherlands) according to the manufacturer’s protocol supplemented with additional acid–ethanol extraction (87.5%v/v EtOH and 2.9% v/v 12 N HCl). An enzymatic calorimetric test was used to measure plasma creatinine (Creatinine PAP FS, DiaSys Diagnostic Systems GmbH, Holzheim, Germany) and serum nonesterified fatty acid (NEFA) (NEFA-HR(2) kit, Wako Chemicals, Neuss, Germany). For the NEFA measurement, different from the manufacturer’s protocol, 5 μL serum was added to the plate and 100 μL of reagent 1 was added to the wells and incubated for 10 min at 37 °C. Subsequently, 50 μL of reagent 2 was added followed by another incubation step of 10 min at 37 °C.

### Follicular fluid IGF1

Follicular fluid IGF1 levels were measured similar to plasma IGF1.

### Statistical analyses

Distributions of the means and residuals were examined to verify model assumptions of normality and homogeneity of variance. Insulin, pregnenolone, β-testosterone, 4-androsten-3-17-dione, and β-nortestosterone levels were log transformed to obtain normality. The presence of outliers was tested by calculating the studentized residuals using proc REG (SAS 9.4, Cary, NC), and outliers were excluded from further analysis. Follicular and metabolic differences between the treatment groups FF and RES were analyzed using proc MIXED in SAS 9.4 (Cary, NC) in models that also contained batch as a random effect. A correction for repeated measures (day of measurement) was added to the model for analysis of body weight, backfat depth, muscle depth, and serum and plasma measurements. Additionally, relations between metabolic parameters and follicular or oocyte parameters were estimated using the model: Yij = μ + treatment + βXij + βXijX^*^treatment + Eijk, where EijkYij is the dependent variable and either a metabolic or follicular or oocyte parameter, β is the regression coefficient, and Xij is one of the metabolic or follicular parameters. The interactions were excluded from the models when not significant. All values are presented as LS means ±SE.

## Results

### Feed intake, metabolic state, and litter growth

The realized feed intake was indeed lower for the RES sows and higher in the FF sows during the feed restriction period (Figure 1). All sows lost body weight during lactation, but RES sows showed a higher percentage loss of their initial body weight (19.6 ± 1.4 vs.11.5 ± 1.4%, *P* < 0.0001) and higher muscle depth loss (1.7 ± 0.1 vs. 1.1 ± 0.1 mm, *P* = 0.01) during lactation as compared to FF sows. RES and FF sows lost a similar amount of backfat during lactation (5.1 ± 0.6 vs. 4.6 ± 0.6 mm for RES and FF, respectively) (Figure 2). Within treatment groups, body weight, muscle depth, and backfat depth significantly decreased during lactation at all measured time points, except for muscle depth between arrival and D1 in the FF sows. Litter growth during lactation was reduced in the litters of the RES sows (56.2 ± 3.9 vs. 66.2 ± 3.9 kg, *P* < 0.01) compared to FF. To further assess the metabolic state of the sows, plasma creatinine, IGF1, insulin, leptin, and serum NEFA levels were measured. Plasma creatinine and IGF1 levels were higher for FF sows at D17 (1 week after start feed restriction), D24 (weaning), and D26, and basal insulin levels were lower for FF sows at D24. Plasma leptin and serum NEFA levels did not differ between FF and RES sows at any of the measured time points (Figure 3).

### Follicle and oocyte developmental competence

**Figure 4:**
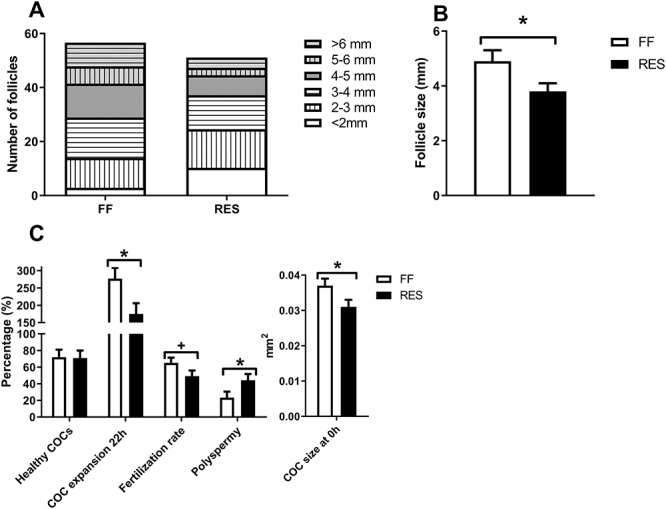
(A) Total number of visible antral follicles in different size categories of both the left and right ovary. (B) Average follicle size of the 15 largest follicles of both the left and right ovary 48 h after weaning and (C) percentage healthy cumulus–oocyte complexes (COCs), average COC size at 0 h, percentage COC expansion at 22 h and fertilization rate of the successfully isolated COCs of the 15 largest follicles of the left ovary, and polyspermy (%) of the fertilized oocytes for full-fed and restricted-fed sows which received either 6.5 or 3.25 kg/day for the last 2 weeks of lactation. COCs were collected 48 h after weaning. ^*^P-value <0.05, ^+^*P*-value ≥0.05 and <0.10.

**Figure 5:**
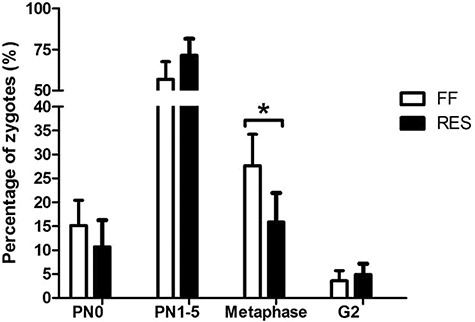
Developmental stage of the presumptive zygotes 24 h after IVF, for full-fed (FF) and restricted-fed (RES) sows, which received either 6.5 or 3.25 kg/day for the last 2 weeks of lactation. Cumulus–oocyte complexes were collected 48 h after weaning. PN0 = pronuclear stage 0, PN1–5 = pronuclear stage 1–5, G2 = 2-cell stage as an average percentage of the total number of zygotes of each animal (total number of zygotes = 6.8 ± 0.6 vs. 4.0 ± 0.6 for FF and RES, respectively). ^*^P-value <0.05.

Total number of visible antral follicles in different size categories of both the left and right ovary is shown in Figure 4A. Average follicle size of the 15 largest follicles of both the left and right ovary was lower for RES as compared to FF sows (Figure 4B). The percentage healthy COCs, as measured at the start of IVM, was similar between the two groups (Figure 4C). However, average COC size at 0 h was smaller, and cumulus expansion was reduced after 22 h of IVM in RES compared to FF (Figure 4C). Oocytes of RES sows tended to have lower fertilization rates (*P* = 0.10), and zygotes showed a higher incidence of polyspermy 24 h after start of IVF (Figure 4C). Fewer of the developing zygotes from RES sows were at the metaphase stage of the first division 24 h after IVF, as compared to zygotes from FF sows (Figure 5). Combined these results suggest a poorer developmental competence of the oocytes that is related to the severity of the NEB during lactation.

**Figure 6:**
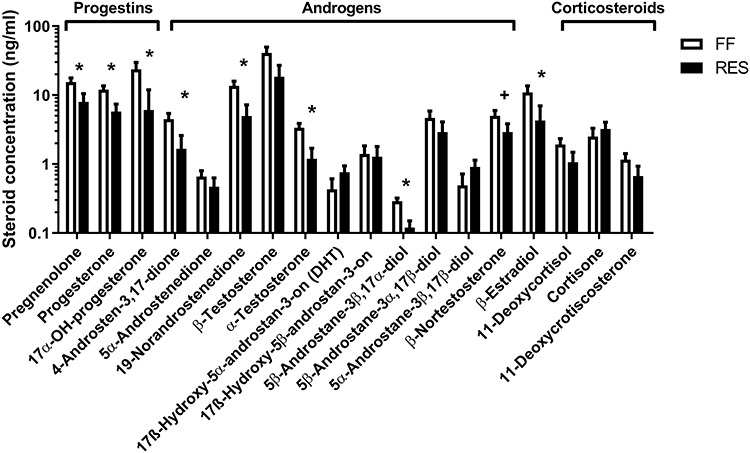
Follicular fluid steroid profile measured 48 h after weaning, of full-fed (FF) and restricted-fed (RES) sows that received either 6.5 or 3.25 kg/day for the last 2 weeks of lactation. ^*^P-value <0.05 ^+^*P*-value ≥0.05 and <0.10.

Pooled follicular fluid of the 15 largest follicles of RES sows had a lower concentration of β-estradiol as compared to FF sows, and follicular fluid of RES sows also had a lower concentration of pregnenolone and intermediates of the delta 4-pathway of steroid production; progesterone, 17α-OH-progesterone, and androstenedione. Intermediates of the delta 5-pathway of steroid production, 17α-OH-pregnenolone, dehydroepiandrosterone (DHEA), and androstenediol were not detectable because of low concentrations. Corticosteroid concentrations did not differ between the two groups (Figure 6 and Supplemental Figure S1 for a schematic overview). Higher β-estradiol and 19-norandrostenedione levels were related to more cumulus expansion 22 h after start of IVM (β = 7.32%/ng/mL, *P* = 0.01 and β = 8.55%/ng/mL, *P* < 0.01, respectively).

**Figure 7:**
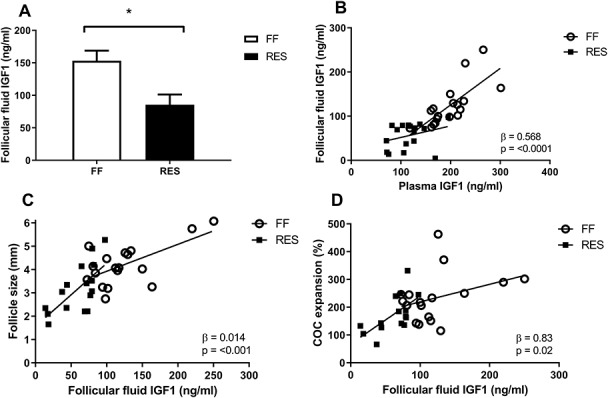
(A) Follicular fluid levels of insulin-like growth factor 1 (IGF1) of full-fed (FF) and restricted-fed (RES) sows that received either 6.5 or 3.25 kg/day for the last 2 weeks of lactation and relation between follicular fluid IGF1 levels (ng/ml) and (B) plasma IGF1 levels at slaughter (48 h after weaning) (C) average follicle size of the 15 largest follicles of both the left and right ovary or (D) COC expansion (%) 22 h after start of IVM of the 15 largest follicles of the left ovary. Ovaries were obtained 48 h after weaning. Relations were corrected for treatment. Interactions with treatment were never significant. ^*^P-value <0.05.

Follicular fluid IGF1 levels were also higher in FF compared to RES sows (Figure 7A). Plasma and follicular fluid IGF1 levels 48 h after weaning were highly correlated (Figure 7B). Higher follicular fluid IGF1 content was further related to a larger average follicle size (Figure 7C) and enlarged COC expansion (Figure 7D).

### Relations between lactational metabolic state of the sows and follicle and oocyte developmental competence

**Figure 8:**
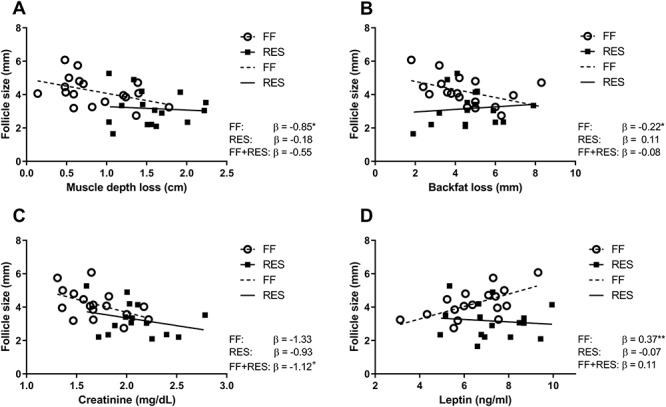
Relations between average follicle size (mm) of the 15 largest follicles of both left and right ovary as measured 48 h after weaning and (A) muscle depth loss (mm) during lactation (B) backfat depth loss (mm) during lactation (C) plasma creatinine (mg/dL) at weaning and (D) plasma leptin (ng/mL) at weaning. Relations were corrected for treatment. ^*^^*^P-value <0.01,^*^P-value <0.05, ^+^*P*-value ≥0.05 and <0.10.

Relations between the lactational metabolic state of the sows and follicular development 48 h after weaning were analyzed, to get a better understanding of the underlying mechanism of metabolic influences on reproductive outcome. Only in FF sows, more lactational muscle depth loss and backfat depth loss were related to a lower average follicle size among the selected 15 largest follicles (Figure 8A and B, respectively). For both FF and RES sows, higher creatinine levels were related to a lower average follicle size (Figure 8C), while for FF sows only, higher plasma leptin levels at weaning were related to a higher average follicle size (Figure 8D). For both FF and RES sows, higher plasma IGF1 levels at weaning were positively related to average follicle size and percentage healthy COCs (*β* = 0.007 mm/ng/mL, *P* < 0.01 and 0.12%/ng/mL, *P* = 0.05, respectively). Finally, relations between follicular and oocyte characteristics were estimated (Supplemental Table S1). A higher average follicle size in the selected pool of 15 largest follicles was related to higher COC expansion rate 22 h after IVM. A larger percentage healthy COCs increased COC expansion rate and higher fertilization rate, as measured 24 h after IVF. Increased COC expansion was related to a higher fertilization rate.

## Discussion

Sows subjected to feed restriction during the last 2 weeks of a 24-day lactation period lost more body weight as compared to sows which were full-fed during the entire lactation period, which as expected, resulted in lower plasma IGF1 levels at weaning. Feed restriction probably also resulted in reduced milk or low-quality milk production, as litter growth was lower in the restricted sows compared to the full-fed sows. During lactation, sows usually mobilize both lean mass and fat mass [19,20]. However, the difference in body weight loss in the current study mainly consisted of lean mass loss, as the restricted sows lost more loin muscle depth and had higher creatinine levels, while the loss of backfat during lactation was similar to those of the full-fed sows. Also, no differences were seen for plasma levels of leptin, a marker for adiposity, at any of the measured time points supporting indicating no effect of feed restriction on fat mass. Lactational feed restriction usually results in higher backfat loss [21–23], although Patterson et al. [8] found similar backfat loss in restricted and full-fed sows. These discrepancies might be related not only to the severity and duration of the feed restriction but also to protein content of the diet and backfat thickness at the start of lactation [24]. In our study, we used TN70 gilts that have a high genetic merit for leanness (personal communication, Louisa Zak, TopigsNorsvin, 2019). Possibly, TN70 sows, when subjected to lactational feed restriction, rapidly mobilize more protein after onset of feed restriction as fat reserves soon become critically depleted. This might explain the lack of differences in lactational backfat loss between the restricted and full-fed sows in the current study.

The main findings of this study are that feed restriction of primiparous sows during the last 2 weeks of lactation negatively impacts follicle size, oocyte developmental competence in vitro, and follicular fluid steroid content 48 h after weaning. The more severe NEB of the feed restricted sows resulted in a smaller average follicle size of the 15 largest follicles. This reduction in follicular growth could be due to an inhibition in luteinizing hormone (LH) pulsatility, which is usually observed in sows as a response to lactational feed restriction [21], as metabolic intermediates influence the neuroendocrine axis in pigs [25]. Subsequently, we investigated if these smaller follicles yielded COCs with a lower developmental competence. One of the ways we assessed this was via morphological classification of COCs as either healthy (intact oocyte and cumulus cell layer) or unhealthy (damaged oocyte or cumulus cell layer), where COCs with intact oocytes and the most cumulus cell layers usually result in the highest maturation and fertilization rates [17,26]. When comparing the two groups of sows in our study, we found that COC morphology was similar between the full-fed and restricted sows. However, the more severe lactational NEB of the restricted sows did result in reduced COC expansion and a tendency in reduced fertilization rate as compared to the more moderate NEB of the full-fed sows. So, although we did not observe differences in COC morphology, the COCs of the restricted sows did show compromised developmental competence. This confirms earlier findings that a more severe lactational NEB negatively impacts in vitro oocyte maturation. For instance, feed restriction for 19 days after oestrus in gilts [27] and feed restriction from day 21 to 28 in a 28-day lactation period in primiparous sows [12] both resulted in a lower level of nuclear oocyte maturation. Different from these studies, we additionally analyzed the developmental stage of the zygotes 24 h after IVF. Zygotes of restricted sows give the impression to be less developed, as fewer zygotes reached the metaphase stage at 24 h after IVF. In addition, a higher percentage of zygotes of restricted sows showed polyspermy. Polyspermy is a common feature of porcine IVF and is considered a marker for oocyte quality, as a higher incidence of polyspermy is seen in immature oocytes, which have not yet completed cytoplasmic maturation [28] and in aged oocytes [29]. Our findings therefore indicate that zygotes of restricted sows have a reduced chance of becoming viable offspring.

Oocyte developmental competence is highly influenced by follicular fluid composition as this determines the microenvironment in which the oocytes develop. To our knowledge, this is the first study to report complete steroid profiles of full-fed and lactational feed restricted sows. Follicular fluid steroid profiling showed that β-estradiol and pregnenolone levels were lower for restricted sows as compared to full-fed sows. In addition, progesterone and androgen intermediates of the Δ4-pathway of steroid production (progesterone, 17α-OH-progesterone, 4-andro-3,17-stenedione, and 19-norandrostenedione) were detected at lower levels in restricted sows as compared to full-fed sows. Intermediates of the Δ5-pathway of steroid production (17α-OH-pregnenolone, DHEA, and androstenediol) were below the detection limit. In human gonads, the Δ5-pathway is the preferred pathway for steroid production [30], which is reflected by the higher efficiency of CYP17A1, involved in the Δ5-pathway as compared to HSD3B which is needed for the conversion of Δ5 into Δ4-steroids [31]. The largely undetectable levels of Δ5-pathway intermediates might be explained by high Δ5-steroidogenic enzyme activity, which would indicate that the Δ5-pathway is also the preferred pathway for follicular steroid production in sows. This is further confirmed by a study, which identified reduced DHEA levels in gilt saliva as a potential biomarker for the start of the waiting period in gilts [32]. The lower steroid levels in the follicular fluid of the restricted sows could at least partially explain the reduced developmental competence of the COCs in restricted sows. This is confirmed by the positive relations between β-estradiol and 19-norandrostenedione levels and COC expansion (%) 22 h after start of IVM. Other studies have found that not only β-estradiol, but also higher progesterone and androgen concentrations, can increase IVM and IVF outcomes when added to the maturation medium of bovine and porcine oocytes [33–35]. Since we did not culture the oocytes in their own follicular fluid, the direct relations between β-estradiol and 19-norandrostenedione and COC expansion as found in our study probably reflect the influence of the steroids on the developmental competence of the COCs before the start of the final maturation during IVM.

We also analyzed follicular fluid IGF1 levels. The restricted sows of our study had lower follicular fluid IGF1 levels as compared to the full-fed sows. Many tissues in anabolic states produce IGF1, including the ovary. Follicular fluid IGF1 levels could therefore be dependent on peripheral IGF1, but also on FSH-induced granulosa cell IGF1 production [36]. In our study, plasma IGF1 levels were highly correlated to follicular fluid IGF levels, suggesting that peripheral IGF1 highly influenced follicular IGF1 levels, either by IGF1 transport from the serum to the follicular fluid, by influencing granulosa cell IGF1 production or both. Follicular IGF1 is essential for follicular development, as it can bind to IGF1 receptors on oocytes and granulosa cells and synergize with FSH to activate the phosphatidylinositol 3-kinase signaling pathway [37] to stimulate follicular growth, steroidogenesis, COC expansion, and oocyte cleavage rate [38–41]. The lower IGF1 levels in the restricted sows could have played an important role in the regulation of reduced follicular growth, steroid production, and oocyte developmental competence.

Finally, we analyzed relations between the measured metabolic and follicular parameters to assess the impact of different metabolic intermediates on follicular development. The restricted sows lost more lean mass but did not differ in the amount of backfat loss during lactation. Despite of this, we observed that feed restriction negatively affected follicular development, as seen by a reduced growth and changed follicular fluid composition, and the developmental competence of the oocytes. It could be speculated that especially lean mass loss negatively impacts follicular development. This is supported by findings of earlier studies, where primiparous sows which lost more lean mass but similar amounts of backfat, induced by different amounts of lysine in their diet, had smaller follicles as measured at weaning [19] or at pro-oestrus [42]. However, in our data, regression analysis showed that next to muscle depth loss, also backfat loss was negatively related to average follicle size, but in full-fed sows only. In addition, higher leptin levels at weaning were related to a higher average follicle size. This indicates that whole-body adiposity and the loss of fat mass during lactation is related to follicular development around weaning. In addition, although the level of lactational backfat loss was similar between the two groups, we found that adipocytes of subcutaneous adipose tissue at slaughter were significantly smaller for restricted sows as compared to full-fed sows (Supplemental Figure S2), indicative of higher lipid mobilization. This is similar to what is found in dairy cows experiencing a lactational NEB [43,44]. The effect of feed restriction on adipocyte size could differ between depots, as based on the measured leptin levels, whole-body lipid mobilisation and adiposity appeared to be similar between the two groups. The influence of lactational feed restriction on adipose tissue functioning of different depots and its relation with follicular development needs to be studied further.

To conclude, feed restriction of sows during the last 2 weeks of lactation negatively impacted follicle size, follicular fluid steroid profile and IGF1 levels, and oocyte and zygote developmental competence. Oocytes with reduced developmental competence originated from follicles with relatively low follicular fluid steroid and IGF1 concentrations. This indicates that the follicles of the restricted sows were less able to produce the necessary steroids and growth factors needed for oocytes to obtain full developmental competence. Finally, results of our study imply that the mobilization of lean mass affects follicular development more than mobilization of fat mass upon feed restriction during lactation, although a reduced fat mobilization positively influenced follicle size in full-fed sows. This knowledge improves our understanding of the relation between energy mobilisation of lean mass vs. fat mass in relation to follicle and oocyte developmental competence. Furthermore, this knowledge could eventually be used for optimal feeding in lactating sows to facilitate a better reproductive outcome.

## Supplementary Material

20190819_nSupplemental_Figure_S2_ioz175

## Notes

Deceased 24-01-2019.
