# Supplementary material for: Consequences of negative energy balance on follicular development and oocyte quality in primiparous sows
Source: Biol Reprod. 2019 Sep 2;102(2):388–98. doi: 10.1093/biolre/ioz175 (PMC7016286; doi:10.1093/biolre/ioz175)
Supplement: 20190819_nSupplemental_Figure_S2_ioz175 [file 20190819_nsupplemental_figure_s2_ioz175.docx]

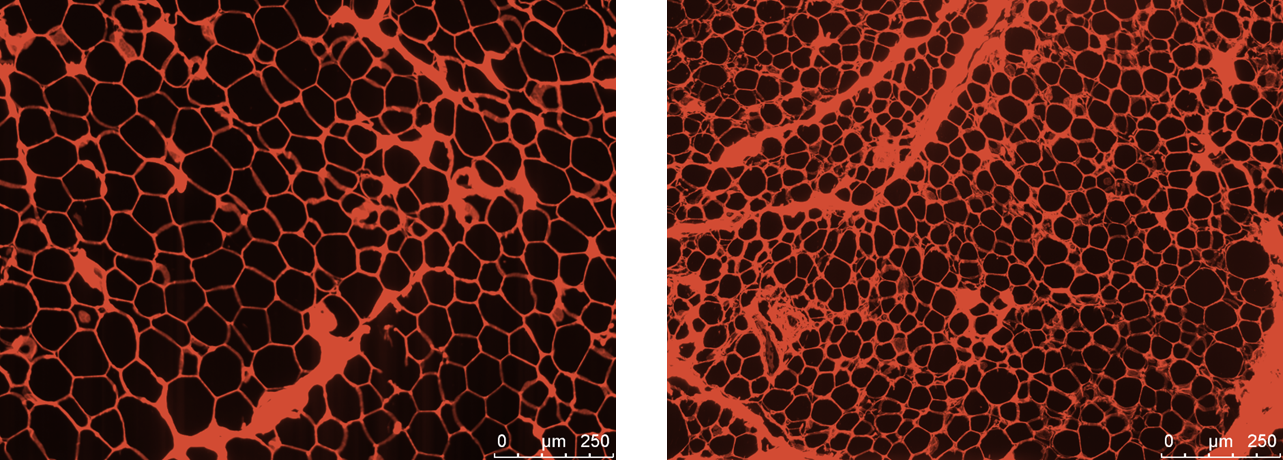


**Supplemental Figure S2** Representative image (100x magnification) of subcutaneous adipose tissue of full-fed (left) and restricted-fed sows which received either 6.5 kg/day or 3.25 kg/day for the last two weeks of a 24-day lactation. Adipose tissue was fixed in 4% formalin and visualised using H&E staining.
